# Estrogen is required for maintaining the quality of cardiac stem cells

**DOI:** 10.1371/journal.pone.0245166

**Published:** 2021-01-22

**Authors:** Al Shaimaa Hasan, Lan Luo, Satoko Baba, Tao-Sheng Li

**Affiliations:** 1 Department of Stem Cell Biology, Atomic Bomb Disease Institute, Nagasaki University, Nagasaki, Japan; 2 Department of Medical Pharmacology, Qena Faculty of Medicine, South Valley University, Qena, Egypt; 3 School of Medical Technology, Xuzhou Medical University, Xuzhou City, Jiangsu Province, China; Augusta University Medical College of Georgia, UNITED STATES

## Abstract

Compared to the age-matched men, the incidence of cardiovascular diseases is lower in premenopausal but higher in postmenopausal women, suggesting the cardio-protective role of estrogen in females. Although cardiac stem cells (CSCs) express estrogen receptors, yet the effects of estrogen on CSCs remain unclear. In this study, we investigated the potential role of estrogen in maintaining the quality of CSCs by *in vivo* and *in vitro* experiments. For the *in vivo* study, estrogen deficiency was induced by ovariectomy in 6-weeks-old C57BL/6 female mice, and then randomly given 17β-estradiol (E_2_) replacements at a low dose (0.01 mg/60 days) and high dose (0.18 mg/60 days), or vehicle treatment. All mice were killed 2 months after treatments, and heart tissues were collected for *ex vivo* expansion of CSCs. Compared to age-matched healthy controls, estrogen deficiency slightly decreased the yield of CSCs with significantly lower telomerase activity and more DNA damage. Interestingly, E_2_ replacements at low and high doses significantly increased the yield of CSCs and reversed the quality impairment of CSCs following estrogen deficiency. For the *in vitro* study, twice-passaged CSCs from the hearts of adult healthy female mice were cultured with the supplement of 0.01, 0.1, and 1 μM E_2_ in the medium for 3 days. We found that E_2_ supplement increased c-kit expression, increased proliferative activity, improved telomerase activity, and reduced DNA damage of CSCs in a dose-dependent manner. Our data suggested the potential role of estrogen in maintaining the quality of CSCs, providing new insight into the cardio-protective effects of estrogen.

## Introduction

Estrogen plays an important role in maintaining the homeostasis of various tissues/organs [1–3]. Estrogen deficiency following menopause has been demonstrated to increase the risk of cardiovascular diseases, osteoporosis, and diabetes [3, 4]. Premenopausal women have a lower incidence of cardiovascular diseases compared to men of similar age, indicating the cardio-protective effects of estrogen [5]. Although multiple observational studies have shown the potency of hormone therapy in preventing cardiovascular diseases [6], recent clinical trials have reported that hormone therapy exhibited no beneficial effect on the outcomes of cardiovascular diseases [1, 7]. These controversial results highlighted the necessity to confirm the reality and further understand the mechanisms underlying the benefit of estrogen in cardiovascular diseases.

Estrogen exerts its biological effects through receptors, including estrogen receptor alpha, estrogen receptor beta, and G-protein-coupled ER, which are present in cardiac myocytes, arterial smooth muscle cells, and endothelial cells [8, 9]. It has been reported that estrogen mediates its cardio-protective effects by regulating lipid metabolism, vessel vasodilation, angiogenesis, oxidative stress, and inflammation [8–12].

Recently, stem cells have been identified in almost all of the tissues/organs, and are known to exhibit a pivotal role in maintaining the homeostasis of the body [13]. Emerging studies have reported that estrogen receptors are expressed in a variety of stem cells, including embryonic stem cells, mesenchymal stem cells, and cardiac stem cells (CSCs) [14–17]. Estrogen can regulate the differentiation of embryonic stem cells [18], inhibit the apoptosis of mesenchymal stem cells [15], and enhance the functional benefit of mesenchymal stem cells for myocardial repair [19].

CSCs, one of the most promising stem cell sources for myocardial repair have been tested in clinical trials and showed beneficial effects [20–27]. It has been demonstrated that E_2_-treated CSCs provide better cardio-protective effects [28]. However, it has not yet been well addressed the role of estrogen in CSCs. Using an ovariectomy (Ovx) estrogen deficiency model in young healthy female mice and *in vitro* experiments, we herein investigated the role of estrogen in maintaining the quality of CSCs.

## Materials and methods

### Animals

For the *in vivo* study, 6-weeks-old female C57BL/6 mice were used. For the *in vitro* study, 10-12-weeks-old female C57BL/6 mice were used. All experiments were approved by the Institutional Animal Care and Use Committee of Nagasaki University, and the experiments were performed following the institutional and national guidelines.

### Ovariectomy and estrogen supplement

The 6-weeks-old female C57BL/6 mice were ovariectomized under general anesthesia. The slow-release E_2_ pellets (Innovative Research of America, FL USA) of a low dose (LE_2_ group: 0.01 mg/60 days, n = 3), or a high dose (HE_2_ group: 0.18 mg/60 days, n = 3) were subcutaneously inserted to the mice soon after ovariectomy [29]. Mice received vehicle treatment following ovariectomy (Ovx group, n = 3) or laparotomy alone (Control group, n = 3) were included for comparisons. The doses of estradiol were selected based on previous studies [29, 30]. All mice were sacrificed at 2 months after treatments. The uterus was excised and weighed, and the atrial tissues were harvested for *ex vivo* expansion of CSCs.

### Ex vivo expansion of CSCs

CSCs were expanded using a method similar to the previously described [31]. In brief, the atrial tissues were minced into small fragments and cultured as “explants” on dishes coated with 15 μg/ml fibronectin (CORNING). Stromal-like flat cells outgrew from the tissue fragments in days and became confluent around 2 weeks after the initiation of culture. The outgrowth cells were collected and counted at 2 weeks and further expanded by general cell passaging. All cultures were done in IMDM basic medium (Gibco) supplemented with 10% fetal bovine serum (HyClone), 100 units/ml penicillin G, and 10 μg/ml streptomycin (WAKO, Japan), at 37°C in a 5% CO_2_ incubator. Twice-passaged CSCs were used for evaluations.

### Characterization of CSCs

The expression of c-kit in CSCs was evaluated by immunostaining as previously described [32]. In brief, twice-passaged CSCs (1×10^4^/well) were cultured in 8-well chamber culture slides (Lab-Tek, Thermo Scientific Nunc) coated with 15 μg/ml fibronectin for 3 days and then fixed with 4% PFA for 10 min. After blocking, the cells were incubated with PE-conjugated rat anti-mouse c-kit antibody (eBioscience). The nuclei were labeled with DAPI. The positively stained cells were counted under a fluorescent microscope with 200-fold magnification in twenty randomly selected fields, and the averages from 3 independent experiments were used for statistical analysis.

### Immunostaining detection of the proliferation, telomerase activity, and DNA damage of CSCs

The proliferative activity, telomerase activity, and DNA damage of CSCs were also detected by immunostaining [32]. In brief, twice-passaged CSCs (1×10^4^/well) were cultured on 8-well chamber culture slides for 3 days and then fixed in 4% PFA for 10 min. After blocking, the cells were incubated with primary antibodies against Ki-67 (Dako), telomerase reverse transcriptase (TERT) (Abcam), and 53BP1 (Abcam), and followed by the appropriate fluorescence-conjugated secondary antibodies. The cell nuclei were labeled with DAPI. Positively stained cells were counted as described above.

### In vitro evaluations on the potential effect of E_2_ supplement to CSCs

We further performed *in vitro* experiments to confirm the potential effect of estrogen on CSCs. Briefly, CSCs were expanded from the atrial tissues of 12-week-old healthy female mice as described above. The same twice-passaged CSCs were cultured in 8-well chamber culture slides with the supplement of 0, 0.01, 0.1, 1.0 μM E_2_ (Sigma-Aldrich) in the medium, as previously described [19, 28]. After 3 days of culture, immunostaining was performed to measure the expression of c-kit, proliferative activity, telomerase activity, and DNA damage in CSCs as described above.

### Statistical analysis

All of the results are presented as the mean±SD. The statistical analysis was performed by using one-way analysis of variance (ANOVA), followed by Tukey’s test as a post comparison between groups (GraphPad Prism). Differences were considered significant when P<0.05.

## Results

### Estrogen supplement attenuated the uterine atrophy in mice after ovariectomy

Estrogen deficiency was induced in six-week-old female mice by ovariectomy, and then given low dose E_2_ (0.01 mg/60 days, LE_2_ group), high dose E_2_ (0.18 mg/60 days, HE_2_ group), or vehicle treatment (Ovx group), respectively. Sham-operated mice were used as healthy controls (Control group). After 2 months of treatments, mice were sacrificed and heart tissues were used for *ex vivo* expansion of CSCs. Compared with the healthy controls, the uterine weight was significantly decreased in ovariectomized mice (9.36±2.17 mg *vs*. 58.46±10.67 mg, P<0.05, S1 Fig), confirmed the estrogen deficiency-induced atrophy. Compared to the Ovx group, the uterine weight was increased significantly in the HE_2_ group (108.23±27.61 mg, P<0.05, S1 Fig), and partially in the LE_2_ group (27.4±10.35 mg, P = 0.16, S1 Fig).

### Estrogen supplement significantly increased the number of CSCs in vivo

**Fig 1:**
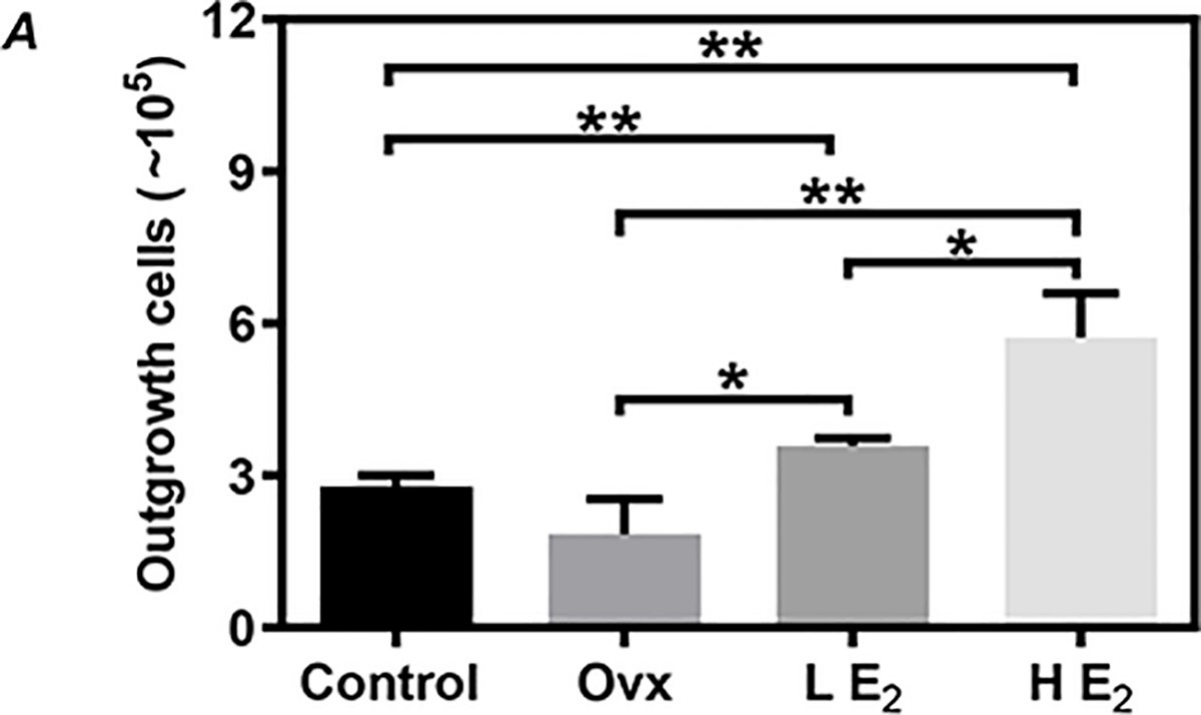
The outgrowth of cardiac stem cells (CSCs) from the atrial tissues of mice. The outgrowth of CSCs was evaluated 2 weeks after the initiation of culture. Control: sham-operated mice, Ovx: ovariectomized mice, LE_2_: ovariectomized mice supplemented with low dose of 17β-estradiol (0.01 mg/60 days), HE_2_: ovariectomized mice supplemented with high dose of 17β-estradiol (0.18 mg/60 days). All data are mean±SD from 3 independent experiments (n = 3 for each group). *P<0.05, **P<0.01.

By using a defined protocol [31], we could easily expand CSCs from atrial tissues from all mice. Although the yield of CSCs was lower in the Ovx group (1.84±0.39 x10^5^ cells) than the Control group (2.78±0.13 x10^5^ cells), there was no significant difference between groups (P = 0.08, Fig 1). Compared with the Ovx group, the yield of CSCs was almost 2-fold higher in the LE_2_ group (3.58±0.08 x10^5^ cells, P<0.05, Fig 1) and 3-fold higher in the HE_2_ group (5.72±0.49 x10^5^ cells, P<0.01, Fig 1).

### Estrogen supplement significantly increased the c-kit expression of CSCs in vivo and in vitro

**Fig 2:**
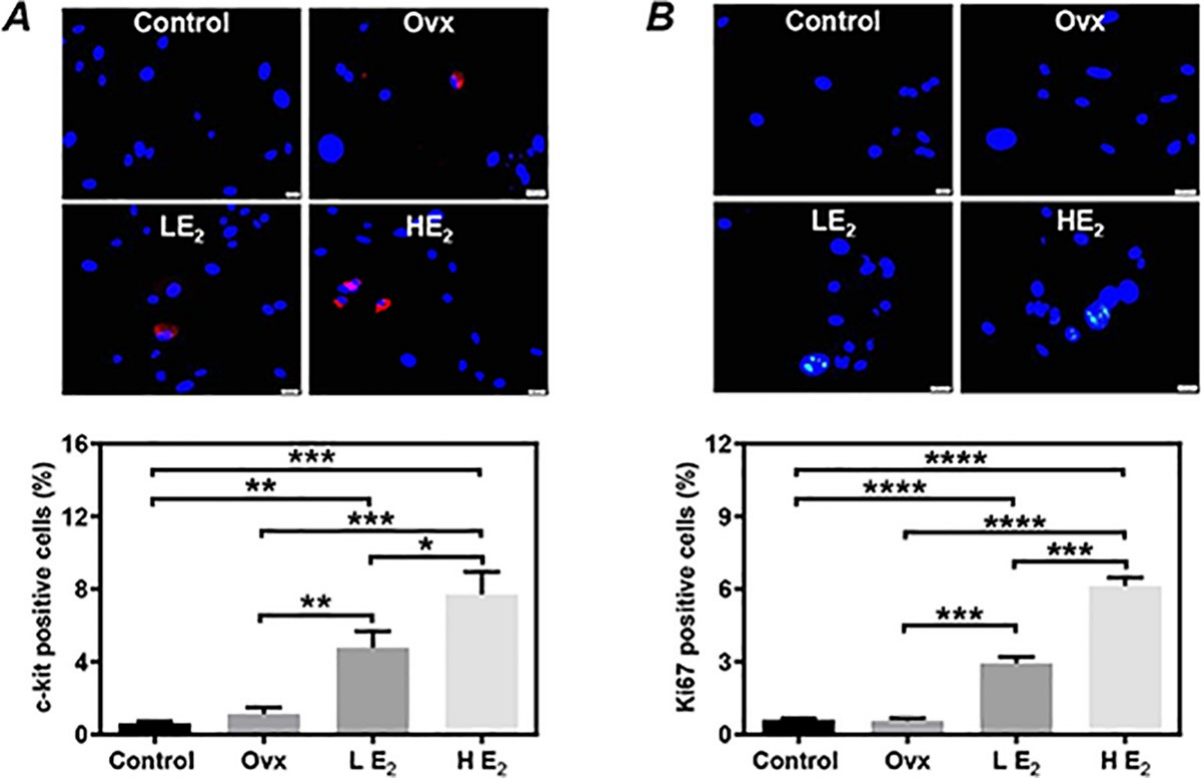
The expression of c-kit and Ki-67 in cardiac stem cells (CSCs). Immunostaining was performed to evaluate the expression of c-kit **(A)** and Ki-67 **(B)** in twice-passaged CSCs. Representative images **(upper)** and quantitative data **(lower)** are shown. The nuclei were labeled with DAPI. Scale bar: 20 μm. Control: sham-operated mice, Ovx: ovariectomized mice, LE_2_: ovariectomized mice received a low dose of 17β-estradiol (0.01 mg/60 days), HE_2_: ovariectomized mice received a high dose of 17β-estradiol (0.18 mg/60 days). All data are mean±SD from 3 independent experiments (n = 3 for each group). *P<0.05, **P<0.01, ***P<0.001, ****P<0.0001.

The expression of c-kit in CSCs was higher in the Ovx group than the Control group, although there was no significant difference between groups (1.13±0.19% *vs*. 0.62±0.05%, P = 0.07, Fig 2A). Interestingly, compared to the Ovx group and the Control group, the expression of c-kit in CSCs was significantly increased in the LE_2_ group (4.77±0.52%, P<0.01, Fig 2A), and even further enhanced in the HE_2_ group (7.71±0.71%, P<0.001, Fig 2A).

**Fig 3:**
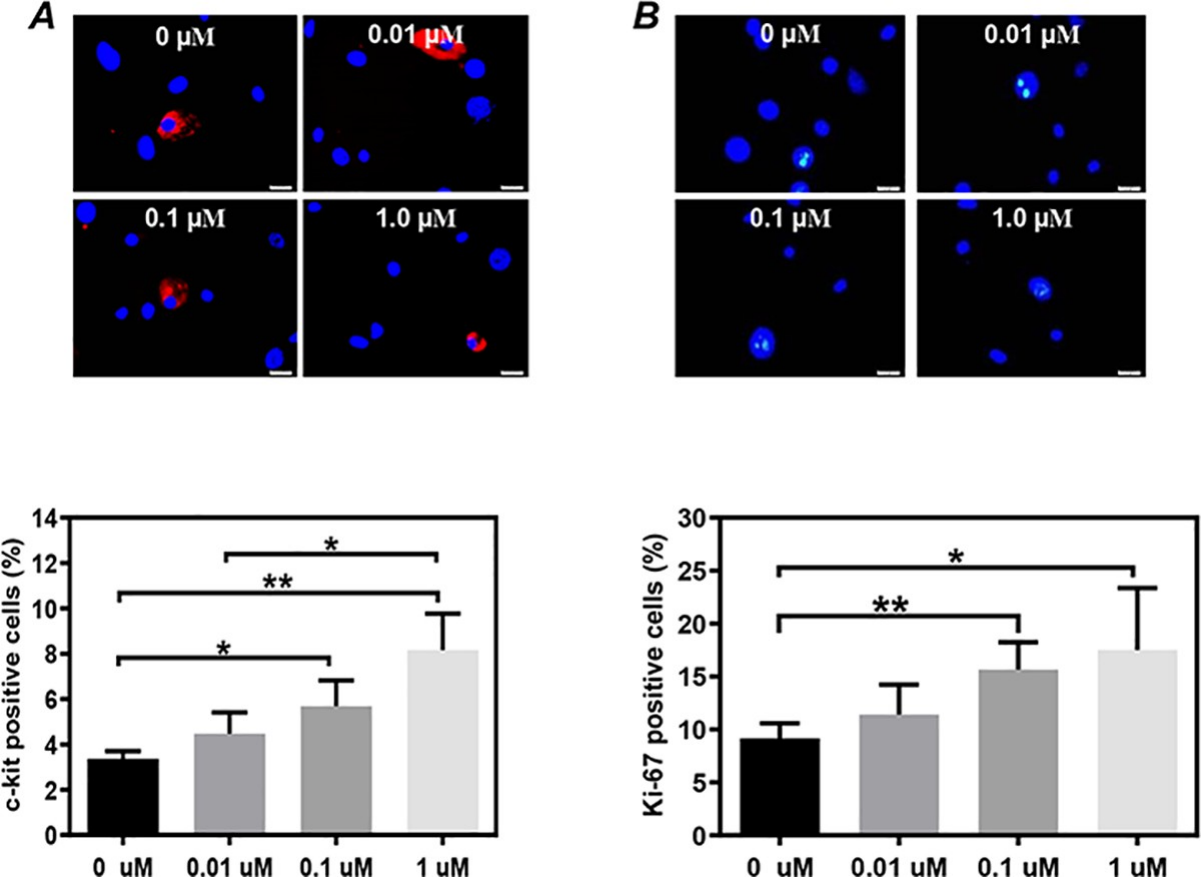
Dose dependency of the 17β-estradiol supplement on the expression of c-kit and Ki67 in cardiac stem cells (CSCs) from healthy female mice. The expression of c-kit **(A)** and Ki-67 **(B)** in CSCs from healthy female mice were evaluated by immunostaining after 3 days of culture with the supplement of different concentrations of 17β-estradiol in the medium. Representative images **(upper)** and Quantitative data **(lower)**. The nuclei were labeled with DAPI. Scale bar: 20 μm. Control: sham-operated mice, Ovx: ovariectomized mice, LE_2_: ovariectomized mice received a low dose of 17β-estradiol (0.01 mg/60 days), HE_2_: ovariectomized mice received a high dose of 17β-estradiol (0.18 mg/60 days). All data are mean±SD from 3 independent experiments (n = 3 for each group). *P<0.05, **P<0.01.

We also evaluated whether the E_2_ supplement would change the expression of c-kit in CSCs from the hearts of healthy female mice *in vitro*. CSCs were cultured with E_2_ supplement at different concentrations for 3 days and then used for analysis. Compared to the vehicle control (3.36±0.19%), the expression of c-kit in CSCs was increased by the supplement with 0.01 μM E_2_ (4.47±0.53%, p = 0.12), 0.1 μM E_2_ (5.68±0.65%, P<0.05, Fig 3A), or 1 μM E_2_ (8.09±0.98%, P<0.01, Fig 3A).

### Estrogen supplement significantly increased the proliferative activity of CSCs in vivo and in vitro

We evaluated the proliferative activity by immunostaining analysis on the expression of Ki-67. The expression of Ki-67 in CSCs did not differ between the Control group and the Ovx group (0.61±0.02% *vs*. 0.56±0.06%, P = 0.49, Fig 2B). However, compared to the Ovx group, the expression of Ki-67 in CSCs was significantly higher in the LE_2_ group (2.94±0.14%, P<0.001, Fig 2B) and the HE_2_ group (6.13±0.19%, P<0.0001, Fig 2B).

We also evaluated whether the E_2_ supplement would change the proliferative activity of CSCs from the hearts of healthy female mice *in vitro*. Compared to the vehicle control (9.15±0.19%), the expression of Ki-67 in CSCs was increased by the supplement with 0.01μM E_2_ (11.42±0.53%, p = 0.2), 0.1 μM E_2_ (15.65±0.65%, P<0.01, Fig 3B), or 1 μM E_2_ (17.51±0.98%, P<0.05, Fig 3B).

### Estrogen supplement significantly improved the telomerase activity and reduced DNA damage of CSCs in vivo and in vitro

**Fig 4:**
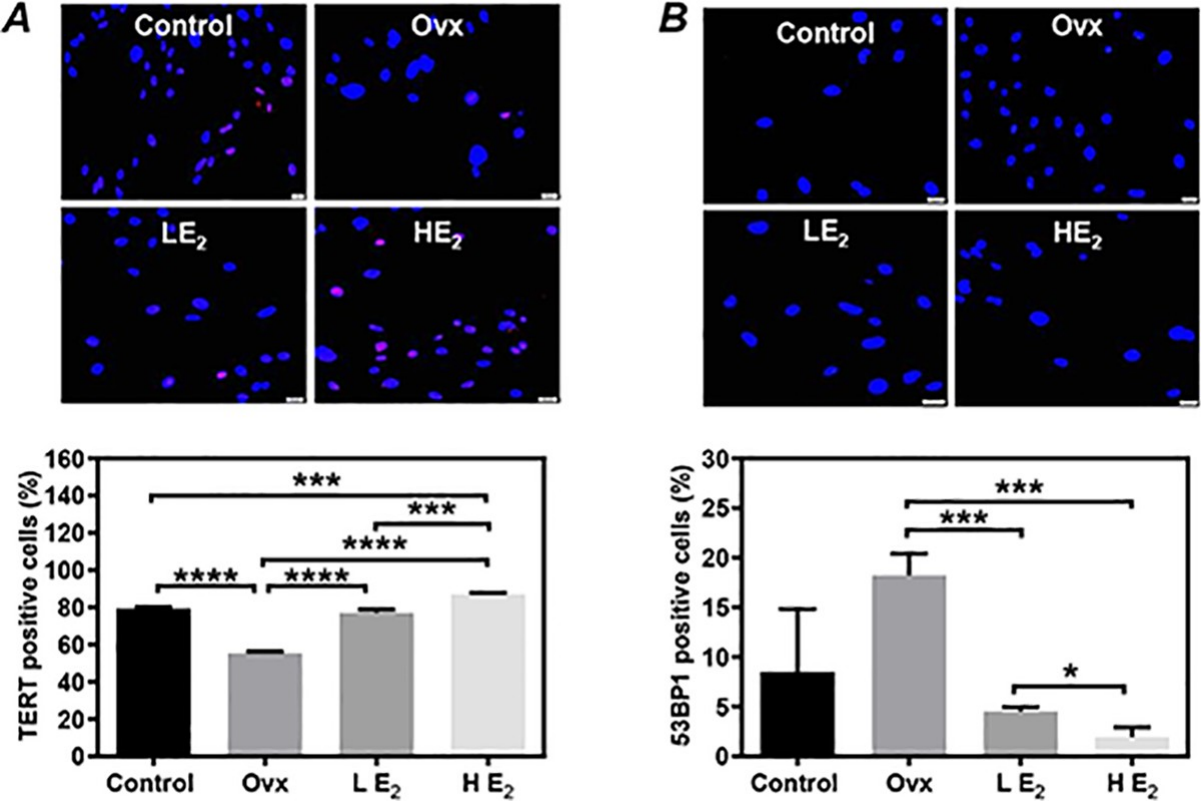
The telomerase activity and DNA damage of cardiac stem cells (CSCs). The telomerase activity and DNA damage in twice-passaged CSCs were evaluated by immunostaining analysis on the expression of TERT **(A)** and 53BP1 **(B)**, respectively. Representative images **(upper)** and quantitative data **(lower)** are shown. The nuclei were labeled with DAPI. Scale bar: 20 μm. Control: sham-operated mice, Ovx: ovariectomized mice, LE_2_: ovariectomized mice received a low dose of 17β-estradiol (0.01 mg/60 days), HE_2_: ovariectomized mice received a high dose of 17β-estradiol (0.18 mg/60 days). All data are mean±SD from 3 independent experiments (n = 3 for each group). *P<0.05, ***P<0.001, ****P<0.0001.

The telomerase activity and DNA damage of CSCs were evaluated by immunostaining analysis on the expression of telomerase reverse transcriptase (TERT) and 53BP1, respectively. Compared to the Control group, the expression of TERT in CSCs was significantly decreased in the Ovx group (55.33±0.58% *vs*. 79.56±0.43%, P<0.0001, Fig 4A), but completely recovered in the LE_2_ group (77.13±1.00%, Fig 4A) and the HE_2_ group (87.12±0.45%, Fig 4A). Although the formation of 53BP1 foci was detected in more CSCs in the Ovx group than the Control group, there was not significant between groups due to the small sample size (18.23±1.25% *vs*. 8.52±3.64%, P = 0.06, Fig 4B). Compared to the Ovx group, the formation of 53BP1 foci in CSCs was detected significantly less in either the LE_2_ group (4.52±0.25%, P<0.001, Fig 4B) or the HE_2_ group (1.94±0.57%, P<0.001, Fig 4B).

**Fig 5:**
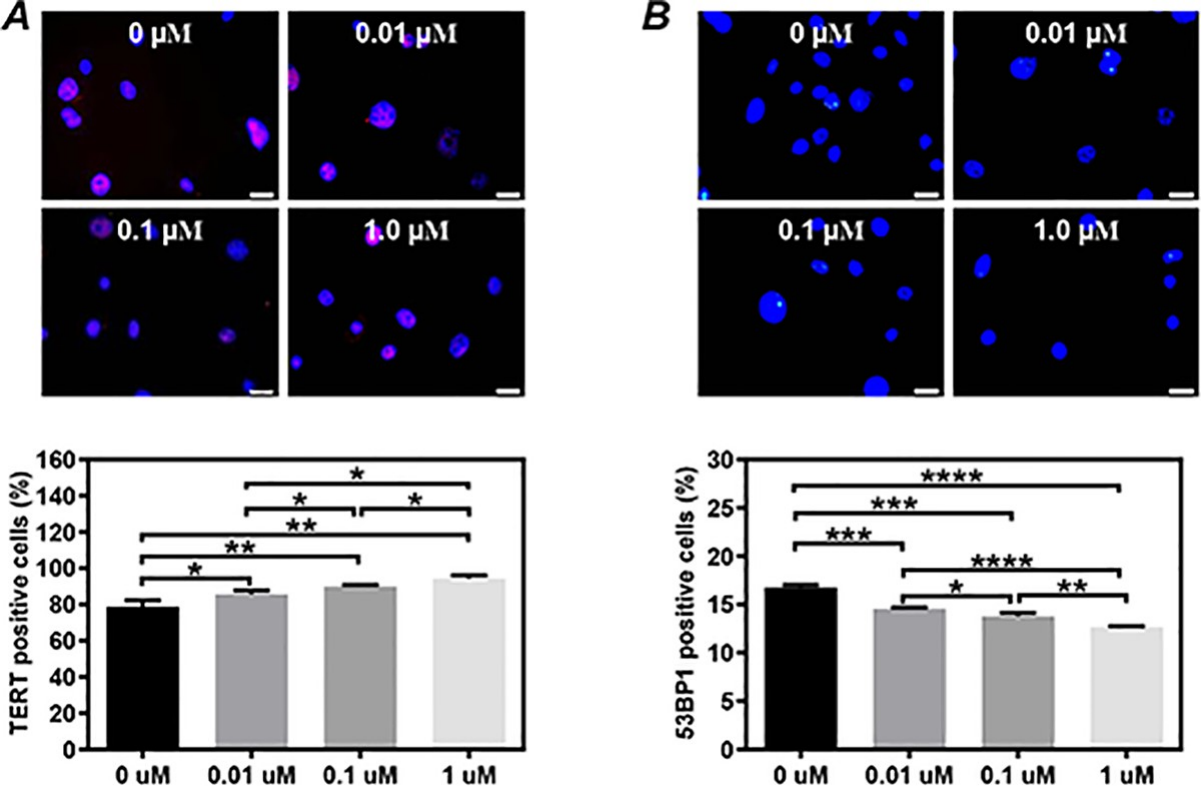
Dose dependency of the 17β-estradiol supplement on the telomerase activity and DNA damage in cardiac stem cells (CSCs) from healthy female mice. The telomerase activity and DNA damage in CSCs were evaluated by immunostaining analysis on the expression of TERT **(A)** and 53BP1 **(B)**, respectively, after 3 days of culture with the supplement of different concentrations of 17β-estradiol in the medium. Representative images **(upper)** and quantitative data **(lower)** are shown. The nuclei were labeled with DAPI. Scale bar: 20 μm. Control: sham-operated mice, Ovx: ovariectomized mice, LE_2_: ovariectomized mice received a low dose of 17β-estradiol (0.01 mg/60 days), HE_2_: ovariectomized mice received a high dose of 17β-estradiol (0.18 mg/60 days). All data are mean±SD from 3 independent experiments (n = 3 for each group). *P<0.05, **P<0.01, ***P<0.001, ****P<0.0001.

We also evaluated the potential effects of E_2_ on telomerase activity and DNA damage of CSCs from the hearts of healthy female mice in vitro. Compared to the vehicle control (78.83±2.03%), the TERT-positive cells in CSCs was increased by the supplement with 0.01μM E_2_ (85.67±1.23%, P<0.05, Fig 5A), 0.1 μM E_2_ (89.96±0.53%, P<0.01, Fig 5A), or 1 μM E_2_ (94.34±0.99%, P<0.01; Fig 5A) in the culture medium. In contrast, compared to the vehicle control (16.75±0.15%), the formation of 53BP1 foci in CSCs was decreased by the supplement with 0.01μM E_2_ (14.55±0.07%, P<0.001, Fig 5B), 0.1 μM E_2_ (13.78±0.21%, P<0.001, Fig 5B), or 1 μM E_2_ (12.65±0.06%, P<0.0001, Fig 5B) in the culture medium.

## Discussion

In the present study, we investigated the potential role of estrogen on CSCs. Using an ovariectomy model in young healthy female mice, we demonstrated that estrogen deficiency-induced quality impairment of CSCs, which could be completely recovered by the replacement with E_2_ following ovariectomy. *In vitro*, the E_2_ supplement in culture medium showed to improve the telomerase and the proliferative activity and reduce the DNA damage of CSCs in a dose-dependent manner. These data suggest that estrogen is likely required for maintaining the quality of CSCs.

Estrogen is known to mediate oxidative stress, inflammatory response, and even the mobilization/recruitment of stem/progenitor cells [8, 28, 33, 34]. However, it is still kept controversial on the beneficial effects of estrogen on the heart. It has been reported that estrogen increases the mortality post-myocardial infarction in mice [35]. In contrast, many studies have demonstrated that estrogen delays the progression of cardiac hypertrophy and heart failure and enhances the functional recovery of infarcted heart in mice and rats [33, 34, 36, 37], but the underlying molecular/cellular mechanisms remain unclear. Therefore, we attempted to investigate the potential role of estrogen on CSCs, a rare population of immature cells known to be critical for maintaining the homeostasis of the heart and regulating the myocardial repair after injury [20–27].

As the beneficial effects of estrogen to the heart may largely depend on the dosage of E_2_ [38, 39], we tested the effects of E_2_ replacement at a low dose (0.01 mg/60 days) and a high dose (0.18 mg/60 days) to mice following ovariectomy. Estrogen deficiency in the ovariectomized mice was confirmed by the presence of uterine atrophy, which was completely recovered by the replacement with high dose E_2_. Although CSCs could be expanded easily from the atrial tissues of all mice received different treatments, estrogen deficiency slightly decreased the yield of CSCs with lower telomerase activity and higher DNA damage. All the estrogen deficiency-induced changes in CSCs could be effectively recovered by the replacement with E_2_ at either low or high doses. The benefits of E_2_ supplement to CSCs were also clearly indicated by various *in vitro* evaluations.

Previous study by Tang et al. has demonstrated the potent regenerative effects of the CSC-derived c-kit-positive cells through paracrine mechanisms [40]. It has also been reported the enhanced cardio-protective effects of CSCs with E_2_ treatment [28]. ERα has been highly expressed on the newly proliferated post-infarct cardiac c-kit+ cells and this receptor stimulated cellular proliferation through activation of the pro-survival PI3K/Akt signaling pathway which enhanced the survival of adult cardiomyocytes following MI [17]. Agreed well with these past studies, our data also clearly showed the benefit of E_2_ in CSCs.

Telomeres are specific DNA-protein components present at the chromosomal terminus of mammalian cells. Telomeres elongation is necessary to prevent DNA damage and senescence for allowing the proliferation of the cells. TERT is responsible for the telomeric replication and maintaining their elongations [41–46]. Estrogen can increase the activity of telomerase through up-regulation of TERT gene [47, 48]. It has been reported that estrogen deficiency inhibits telomerase activity and decrease the proliferation of cells [49, 50]. Otherwise, aging and pathological conditions have been found to decreases the number and quality of CSCs [51, 52]. Considering the pivotal role of estrogen on the maintenance of CSCs, the cardio-protective effects of estrogen may depend, at least partially on the dynamic regulation of CSCs. As we only investigated the quality of CSCs from intact hearts of young mice, it is still unclear whether and how estrogen mediates the CSCs to repair a damaged heart, especially in aged individuals and patients complicated with other clinical pathophysiological conditions.

There are several limitations to this study. First, we only used female mice for experiments and did not measure the E_2_ levels in mice. Although the bioassay method (e.g. uterine weight) may help to monitor the long-term effects of estrogen replacement using the estradiol-sustained-release E_2_ pellets, it is asked to identify the optimal dose of E_2_ supplementation to keep the estrogen at a physiological level for mice received ovariectomy. Second, the adverse effects of high-dose estrogen on other organs needed to be evaluated. Third, we did not measure the cardiac hemodynamics or the cardiac function using a damaged heart as an experimental model, and the telomerase activity was only identified by immunostaining on the expression of TERT. Otherwise, we did not try to further identify the underlying molecular mechanisms on how estrogen maintaining the quality of CSCs.

In summary, our data indicated the potential role of estrogen in maintaining the quality of CSCs, which may provide a novel insight into the cardio-protective effects of estrogen. Uncovering the relevant molecular mechanisms may help to develop specific pharmacological compounds that selectively target the estrogen receptors for the treatment/prevention of postmenopausal cardiovascular diseases.

## Supporting information

S1 Fig Uterine weight of mice. Uterine weight was measured 2 months after treatments. Control: sham-operated mice, Ovx: ovariectomized mice, LE_2_: ovariectomized mice supplemented with low dose of 17β-estradiol (0.01 mg/60 days), HE_2_: ovariectomized mice supplemented with high dose of 17β-estradiol (0.18 mg/60 days). n = 3 for each group. All data are mean ± SD from 3 independent experiments. *P<0.05. The statistical significance was determined by one-way analysis of variance (ANOVA), followed by Tukey’s test as a post comparison between groups (GraphPad Prism).
(JPG)

S1 Table *In vivo* raw data. (XLSX)
