# Supplementary figures and images for: Estrogen is required for maintaining the quality of cardiac stem cells
Source: PLoS One. 2021 Jan 22;16(1):e0245166. doi: 10.1371/journal.pone.0245166 (PMC7822545; doi:10.1371/journal.pone.0245166)

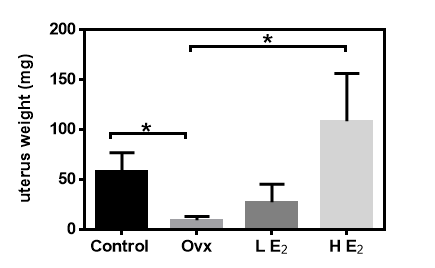

Supplement: S1 Fig — Uterine weight was measured 2 months after treatments. Control: sham-operated mice, Ovx: ovariectomized mice, LE2: ovariectomized mice supplemented with low dose of 17β-estradiol (0.01 mg/60 days), HE2: ovariectomized mice supplemented with high dose of 17β-estradiol (0.18 mg/60 days). n = 3 for each group. All data are mean ± SD from 3 independent experiments. *P<0.05. The statistical significance was determined by one-way analysis of variance (ANOVA), followed by Tukey’s test as a post comparison between groups (GraphPad Prism). (JPG) [file pone.0245166.s001.jpg]
